# An Antisweat Interference and Highly Sensitive Temperature Sensor Based on Poly(3,4-ethylenedioxythiophene)–Poly(styrenesulfonate) Fiber Coated with Polyurethane/Graphene for Real-Time Monitoring of Body Temperature

**DOI:** 10.1021/acsnano.3c04246

**Published:** 2023-10-24

**Authors:** Wei Fan, Tong Liu, Fan Wu, Shujuan Wang, Shengbo Ge, Yunhong Li, Jinlin Liu, Haoran Ye, Ruixin Lei, Chan Wang, Qiuling Che, Yi Li

**Affiliations:** † School of Textile Science and Engineering, Key Laboratory of Functional Textile Material and Product of Ministry of Education, Institute of Flexible Electronics and Intelligent Textile, Xi’an Polytechnic University, Xi’an 710048, China; ‡ School of Chemistry, Xi’an Jiaotong University, Xi’an 710049, China; § College of Materials Science and Engineering, Nanjing Forestry University, Nanjing, Jiangsu 210037, China; ∥ ANTA (China) Co., Ltd., Quanzhou 362000, China; ⊥ Department of Materials, University of Manchester Oxford Road, Manchester M13 9PL, United Kingdom

**Keywords:** temperature sensor, smart wearable devices, PEDOT:PSS fiber, graphene, real-time monitoring, wireless transmission

## Abstract

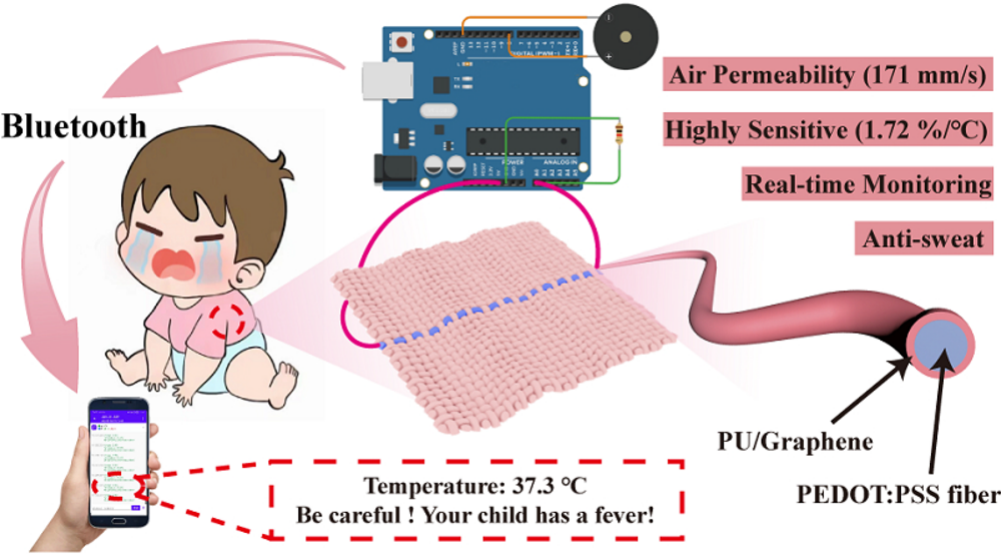

Body temperature is an important indicator of human health. The traditional mercury and medical electronic thermometers have a slow response (≥1 min) and can not be worn for long to achieve continuous temperature monitoring due to their rigidity. In this work, we prepared a skin-core structure polyurethane (PU)/graphene encapsulated poly(3,4-ethylenedioxythiophene)–poly(styrenesulfonate) (PEDOT:PSS) temperature-sensitive fiber in one step by combining wet spinning technology with impregnation technology. The composite fiber has high sensitivity (−1.72%/°C), super-resolution (0.1 °C), fast time response (17 s), antisweat interference, and high linearity (*R*^2^ = 0.98) in the temperature sensing range of 30–50 °C. The fiber is strong enough to be braided into the temperature-sensitive fabric with commercial cotton yarns. The fabric with good comfort and durability can be arranged in the armpit position of the cloth to realize real-time body temperature monitoring without interruption during daily activities. Through Bluetooth wireless transmission, body temperature can be monitored in real-time and displayed on mobile phones to the parents or guardians. Overall, the fiber-based temperature sensor will significantly improve the practical applications of wearable temperature sensors in intelligent medical treatment due to its sensing stability, comfort, and durability.

## Introduction

Body temperature refers to the internal temperature of the body. A relatively constant body temperature is one of the important conditions for maintaining normal human life activities.^1^ In normal people, the armpit temperature is 36.2–37.2 °C.^2^ Body temperature that exceeds 41 °C or falls below 25 °C can have severe consequences for the functioning of all bodily systems, particularly the nervous system, and may even threaten life. For example, 21 participants died of hypothermia caused by a sudden weather change during the 2021 Marathon 100 km cross-country race in Gansu, China. The normal regulation of body temperature can be impaired by various diseases, resulting in changes in body temperature.^3^ Therefore, it is essential to closely monitor body temperature and observe any changes to aid in the diagnosis and prevention of certain illnesses.

Body temperature can be measured by oral measurement, axillary measurement, and anal measurement. The axillary method represents the most widely used method due to its simplicity. However, the traditional mercury and medical electronic thermometer used to test the underarm temperature has a slow response (≥1 min). It is generally known that the smaller the response time of the temperature sensor, the faster it can provide feedback on human body temperature, facilitating rapid medical treatment.^2^ Besides, these thermometers cannot be worn for a long time to achieve continuous temperature monitoring because of their rigidity.^4^ Flexible temperature sensors have gained significant attention since the introduction of flexible wearable technology because of their flexibility and good interface compatibility.^5^ However, most of the present flexible temperature sensors are constructed on impermeable film substrates such as PDMS and PET,^6,7^ which can lead to sweat accumulation on the human skin when attached for an extended period and may even cause skin inflammation in extreme cases.^8,9^ Due to the flexibility, breathability, wearability, and good compatibility with textiles, fiber-based temperature-sensors can detect fever, wound healing, and cardiovascular disease in real-time,^10,11^ which is useful for those who are incapable of speaking or feeling for themselves such as infants, deaf patients, and elderly patients who have Alzheimer’s disease.^12^

The temperature sensing materials of temperature-sensitive fibers include metals (silver, copper, and platinum, etc.),^4^ carbon-based materials (carbon nanotubes (CNTs), graphene oxide (GO), and reduced graphene oxide (rGO), etc.),^13,14^ and conductive polymers (poly(3,4-ethylenedioxythiophene)–poly(styrenesulfonate) (PEDOT:PSS), and polyaniline (PANI), etc.).^15,16^ For example, Trung et al. developed a fiber-based temperature sensor with a sensitivity of −0.8%/°C, a response time of 7 s and a resolution of 0.1 °C by wet spinning rGO and polyurethane (PU) together.^17^ Zhao et al. printed rGO fiber-based temperature sensors with a sensitivity of −1.95%/°C, a response time of 2 s and a resolution of 0.4 °C.^18^ Typically, an ideal human sensor would provide high sensitivity and short response times at a resolution of at least 0.1 °C. However, current fiber-based temperature sensors are not up to the ideal level. PEDOT:PSS has a negative temperature coefficient, high linearity (≥0.97), excellent conductivity (up to 6200 S/cm), and good biocompatibility, which is an ideal material for the preparation of fiber-based temperature sensors.^19^ However, PEDOT:PSS fiber as a kind of polymer is greatly affected by humidity, so it can easily cause inaccurate measurements after human sweating.^20^ Therefore, developing a fast response, antisweat interference, and high-resolution PEDOT:PSS temperature-sensitive fiber is urgently needed for practical sensing applications.

PEDOT:PSS fiber is often prepared by wet-spinning using acetone or isopropanol (IPA) as coagulation baths.^21^ However, fibers prepared using a single coagulation bath exhibit subpar temperature sensing performance due to their low electrical conductivity.^22,23^ Fortunately, dimethyl sulfoxide (DMSO) can increase the electrical conductivity of PEDOT:PSS by reducing the coulomb interaction between PEDOT and PSS and increasing the concentration of charge carriers.^24,25^ It was found that the impact of DMSO concentration on the electrical conductivity of the PEDOT:PSS fiber has yet to be investigated.^24^ PU is a waterproof material that can be coated on conductive material to avoid moisture impacting its conductivity,^26^ but PU has poor thermal conductivity, which may impede body temperature transmission to temperature-sensitive fibers inside. On the other hand, graphene has excellent thermal conductivity and a negative temperature coefficient, which can be added to PU to improve its thermal conductivity.^27^ Therefore, it is hypothesized that PU with a graphene coating on the outside of the fiber will effectively protect against moisture and quickly conduct body temperature to the inner fiber.

In this work, we developed a PU/graphene encapsulated PEDOT:PSS temperature-sensitive fiber (composite fiber) with a skin-core structure in one step by combining wet spinning technology with impregnation technology. The sensitivity, resolution, time response, linearity, and antisweat interference of the composite fiber in the temperature sensing range of 30–50 °C were investigated. The composite fibers were then woven with cotton yarns to form a temperature-sensitive fabric. An assessment was conducted to establish if the fabric is appropriate for daily use considering its comfort and durability. Furthermore, we have successfully designed a Bluetooth module that enables the fabric to monitor body temperature and relay the information to concerned parties or guardians via phone. Fiber-based temperature sensors have potential benefits in various scenarios, including temperature detection and disease prevention in epidemic diseases like COVID-19. Moreover, it could also be useful for long-distance runners and workers in specific occupations such as undersea workers to monitor their body temperature for safety and health purposes.

## Results and Discussion

### Fabrication of PU/Graphene Encapsulated PEDOT:PSS Fiber

**Figure 1:**
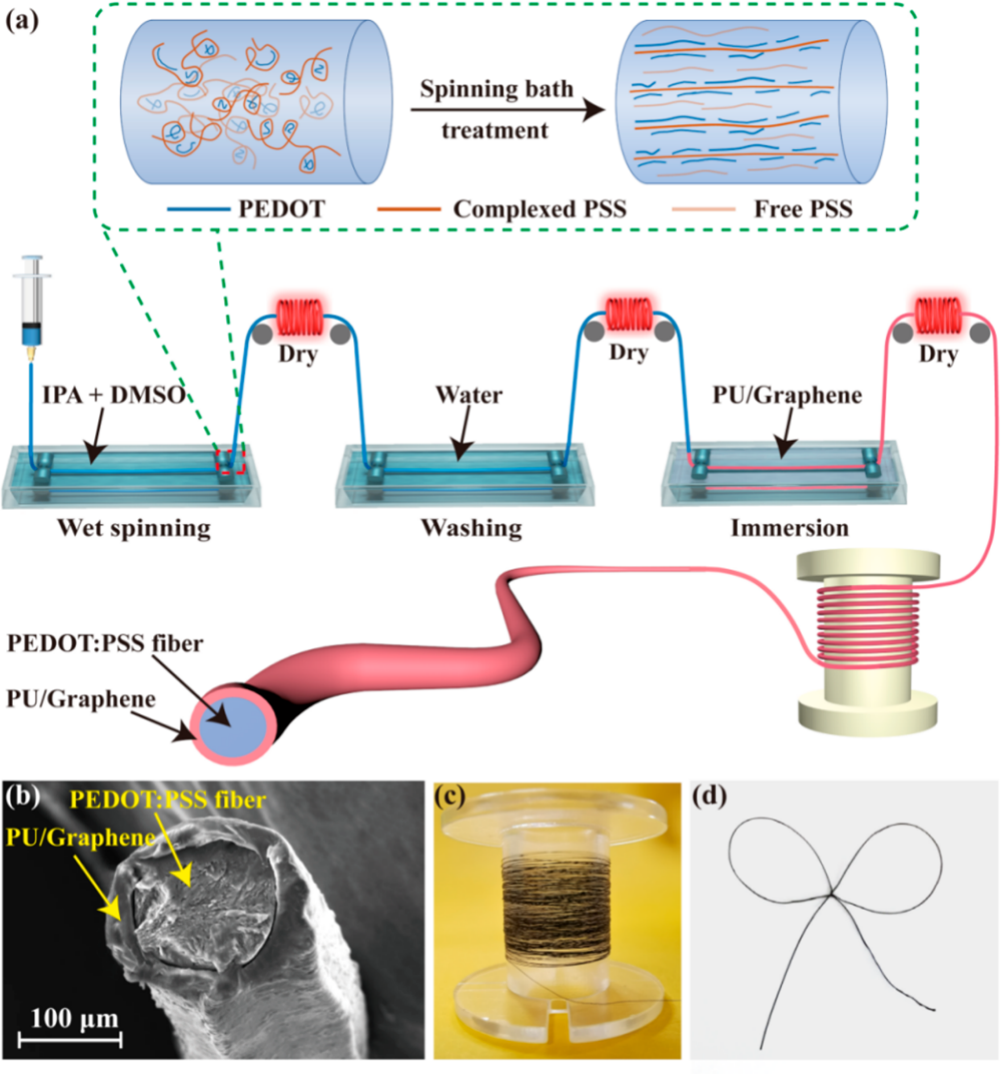
Preparation and characterization of PU/graphene encapsulated PEDOT:PSS fiber (composite fiber) with skin-core structure. (a) Preparation diagram of the composite fiber. (b) SEM image of the composite fiber cross-section. (c) Composite fibers packaged on the reel; (d) knotted composite fibers.

Figure 1a shows the fabrication process for PEDOT:PSS fibers prepared by wet spinning. First, 0.263 g of PEDOT:PSS particles were added into 5 mL of deionized (DI) water and then stirred at 500 r/min for 30 min, and the mixed solution was used as the spinning solution. The mixture of isopropanol (IPA) and dimethyl sulfoxide (DMSO) (1:1, 2:1, and 3:1 by volume) was used as coagulation baths. Then, the spinning solution was extruded into the coagulation bath using a syringe pump at 5 mL/h to form the as-spun PEDOT:PSS fiber. After drying, washing in DI water, and drying again, the fiber was immersed in a mixed solution of PU and graphene to form the PU/graphene encapsulated PEDOT:PSS fibers with a skin-core structure (graphene concentrations of 5, 10, and 15 wt %). Finally, the composite fiber was dried for subsequent use. The surface of PEDOT:PSS fiber is uniformly coated with a PU/graphene layer and the diameter changes from 120 to 150 μm after the encapsulation (Figure 1b). Figure 1c shows the optical picture of the composite fiber packaged on the reel. The composite fiber with good flexibility can be bent and knotted arbitrarily without breaking (Figure 1d).

### Temperature-Sensing Performance of the Pure PEDOT:PSS Fiber

**Figure 2:**
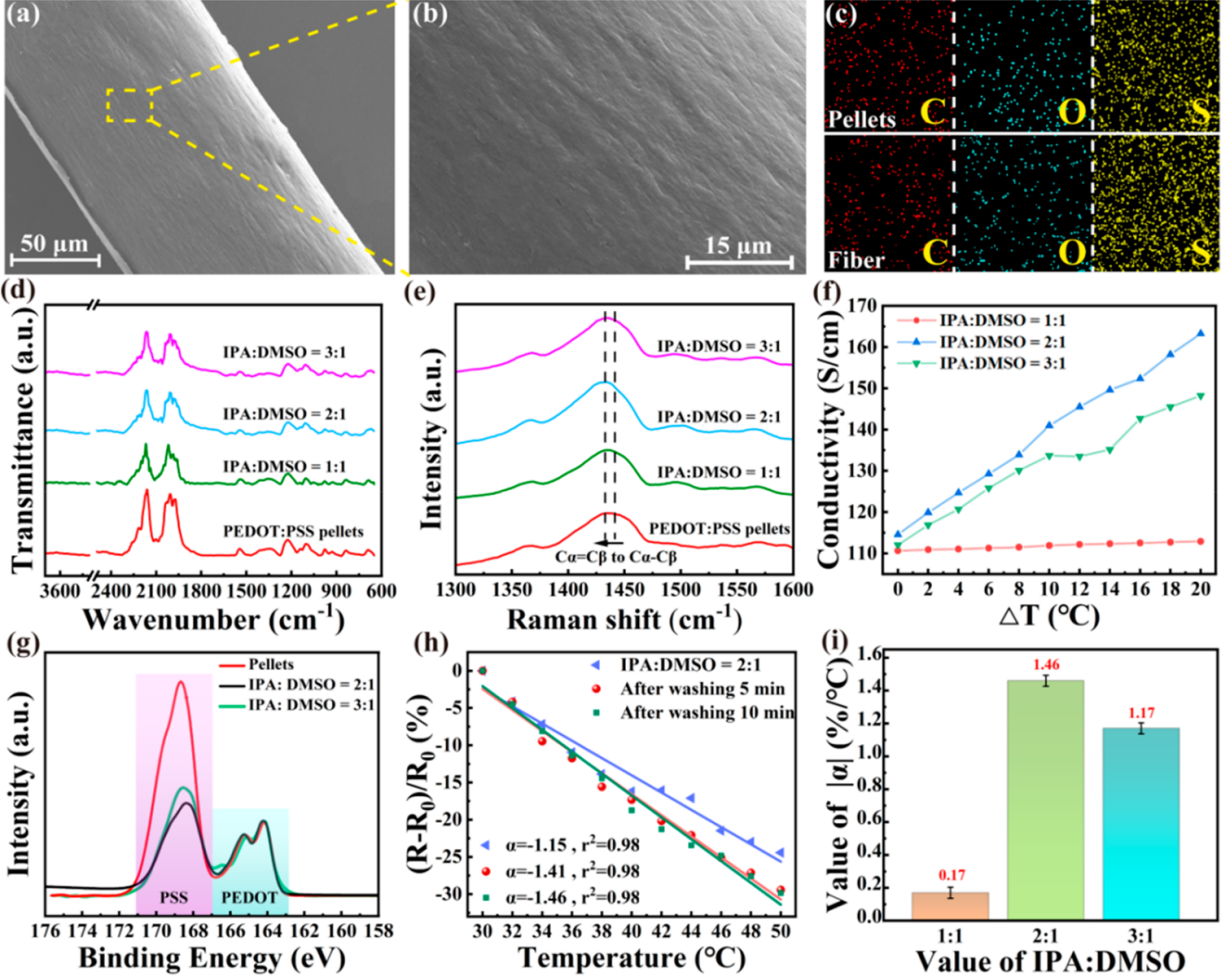
Performance of the PEDOT:PSS fiber: (a) SEM image of the fiber; (b) magnified image of the fiber; (c) EDS images of the PEDOT:PSS pellets and its fiber; FTIR spectra (d) and Raman spectra (e) of PEDOT:PSS pellets and the fibers treated with different coagulation baths; (f) electrical conductivity of the fibers treated with different coagulation baths; (g) XPS spectra of PEDOT:PSS pellets and the fibers at 2:1 and 3:1 of IPA/DMSO; (h) temperature-sensing performance of the fiber (IPA/DMSO = 2:1) before and after washing different times in water; (i) temperature-sensing sensitivity of the fibers under different coagulation baths treatment.

Figure 2a,b show SEM images of the PEDOT:PSS fiber. There are some small grooves on the surface of the fiber. These grooves are caused by the fast diffusion of coagulation baths to as-spun PEDOT:PSS fibers during solidification. Figure 2c depicts the EDS analysis of the PEDOT:PSS pellets and fiber. The results indicate that the elemental composition of the fiber remains unchanged after undergoing coagulation bath treatment. The composition of the fiber still consists of C, O, and S, which is similar to the composition of the original PEDOT raw material without elemental changes. Figure 2d shows the FTIR spectra of PEDOT:PSS pellets and fibers solidified at different coagulator ratios. The peaks at 685, 830, and 984 cm^–1^ are associated with the C–S bond stretch vibrations in the thiophene ring, whereas the peaks at 1519 and 1278 cm^–1^ are assigned to the C=C and C–C tensile vibrations of the thiophene ring.^28^ The C–O–C bond could be inferred from the peaks at 1025, 1065, and 1163 cm^–1^, while the band presented at 2100 cm^–1^ could be ascribed to vibrations of the CO_2_ molecules.^29^ The locally enlarged FTIR spectra are shown in Figure S1. Overall, the FTIR spectral results indicate no chemical reactions during fiber spinning.

Raman spectra of the PEDOT:PSS pellets and their fibers solidified in different coagulator ratios are shown in Figure 2e. The peaks at 1362 cm^–1^ are attributed to the C_β_–C_β_ stretch of PEDOT. The relatively obvious absorption peak at 1442 cm^–1^ could be caused by the C_α_=C_β_ symmetrical contraction vibration of a single five-membered thiophene ring on the PEDOT main chain.^23^ After the coagulation bath treatment, the characteristic peak position is red-shifted from 1442 to 1432 cm^–1^, which can be attributed to the transformation of C_α_=C_β_ (benzene type) connecting thiophene rings in PEDOT to C_α_–C_β_ (quinone type) under the coagulation bath treatment.^23^ The molecular structures of the benzene and quinone types are shown in Figure S2. It was found that the benzene-type structure indicates a curly PEDOT chain, while the quinone-type structure indicates a linear PEDOT chain. The findings revealed that quinone types provide a higher degree of order in PEDOT:PSS molecules than benzene types and thus facilitate the migration of carriers and enhance fiber conductivity after coagulation.^30^

Figure 2f illustrates the conductivities of the fibers treated with different coagulation baths at different temperatures. It was found that with increasing the ratio of IPA and DMSO in the coagulation bath, the conductivity of the fiber increases at first but then decreases. When the ratio is 2:1, the conductivity of the fiber varies most significantly with temperature. Since PSS contributes to the low electrical conductivity of the fibers, removing PSS from PEDOT:PSS can improve their electrical conductivity.^31^ The ratio of PEDOT to PSS can be reflected by the ratio of the sulfur atoms in thiophene and sulfonate, which can be seen in the XPS spectra of the PEDOT:PSS pellets and their fibers (Figure 2g). The lower binding energy peaks at 163.7 and 164.8 eV belong to the sulfur atoms in the thiophene ring (PEDOT), while the broad peaks at 166–170 eV belong to the sulfur atoms in the sulfonate (PSS).^32,33^ For PEDOT:PSS pellets, the ratio of thiophene to sulfonate is 1:3.29, while the values become 1:1.653 and 1:1.584 when the coagulator ratio is 3:1 and 2:1, respectively. The sulfur content in PEDOT has no obvious change, but that in PSS decreases dramatically, indicating that PSS is well removed under the coagulation bath treatment. The separation of PSS can change the curly PEDOT molecular chain to a linear structure, making the transmission of charge carriers easier, thus causing changes in resistance and improving the temperature-sensing sensitivity of the fiber.^24^ Therefore, the optimum ratio of IPA to DMSO for subsequent use is 2:1.

Figure 2h shows the temperature-sensing performance of the PEDOT:PSS fibers (IPA/DMSO = 2:1) before and after different water washing times at the 30–50 °C. The insulating PSS around the PEDOT shrinks with increasing temperature and the distance between adjacent PEDOTs decreases, which increases the electron hopping between PEDOTs.^6^ The sensitivity of the temperature sensor refers to the response degree of an electrical signal to temperature changes.^4^ If the temperature sensor has a high sensitivity, then the response to temperature changes is fast and accurate. The temperature coefficient of resistance (TCR) of material is generally used to determine the sensitivity of temperature sensors as follows:
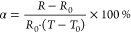
where *T*_0_ is the initial temperature, *T* represents the real-time temperature, *R*_0_ denotes the material resistance at the initial temperature, and *R* refers to the real-time resistance. The α value of the PEDOT:PSS fiber (IPA/DMSO = 2:1) is −1.15%/°C with a linearity of 0.98. The α value of the fiber increased from −1.15%/°C to −1.41%/°C after it was washed for 5 min in water and tended to be stable (−1.46%/°C) after being washed for 10 min. The reason behind this is that washing the fiber with water eliminates residual coagulation bath and insulating PSS on its surface, thereby enhancing its conductivity.^34^Figure 2i compares the temperature-sensing performance of the PEDOT:PSS fibers treated by different coagulation baths. When the ratio of IPA to DMSO is 2:1, the fiber exhibits the highest sensitivity, which is consistent with the results shown in Figure 2f.

### Temperature-Sensing Performance of PU/Graphene Encapsulated PEDOT:PSS Fiber

**Figure 3:**
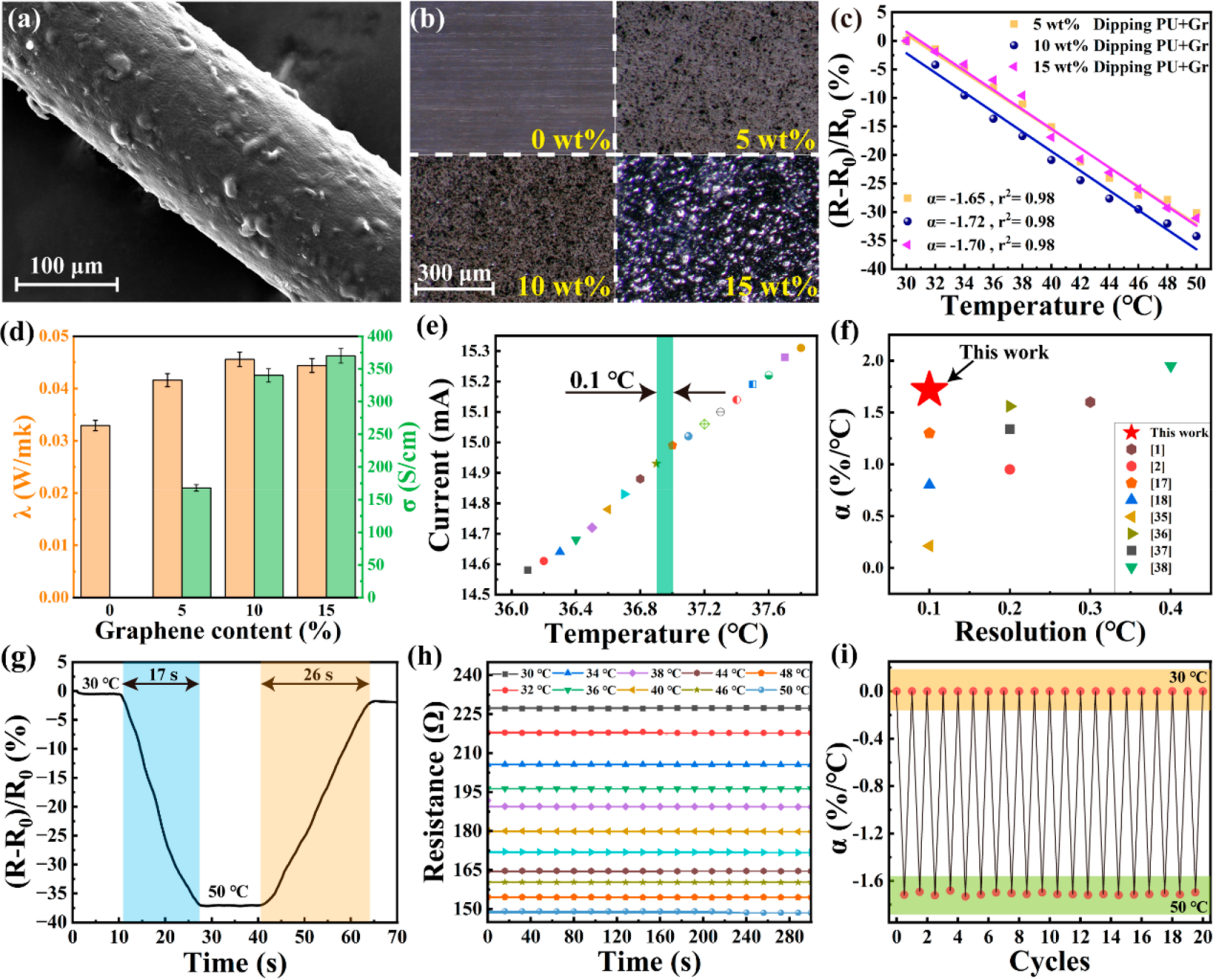
Characterization and sensing performance of the composite fibers: (a) SEM image of the composite fiber; (b) ultradepth microscope pictures of the PU/graphene films with different graphene contents; (c) temperature sensitivity of the composite fibers with different graphene contents; (d) thermal conductivity (λ) and electrical conductivity (σ) of the PU/graphene films with different graphene contents; (e) the current curves of the composite fibers in the temperature range of 36.1–37.8 °C at a 0.1 °C increment; (f) temperature-sensing sensitivity and resolution comparison results between the composite fiber and other flexible temperature sensors ; (g) response time of the composite fiber; (h) resistance stability of the composite fiber at different temperatures; (i) cycle stability of the composite fiber.

The SEM image and of the composite fiber (Figure 3a) and the Raman spectra of PU, graphene, and the composite fiber surface (Figure S3) show that the PU/graphene has been successfully impregnated on the PEDOT:PSS fiber surface. In order to observe the distribution of graphene in PU solution, the ultradepth field image of the PU/graphene mixture with the same thickness is shown in Figure 3b. The surface coarseness tends to be obvious with increasing graphene content. When the graphene content is 15 wt %, the gap between graphene becomes large, suggesting that the graphene is not uniformly dispersed and presents an agglomeration state. When the graphene content is 10 wt %, the α value can reach −1.72%/°C with a linearity of 0.98, which is 1.18 times the sensitivity of the unpackaged PEDOT:PSS fiber (Figure 3c).

In Figure 3d, the conductivity of the PU/graphene layer increases with the graphene content. This is because the highly conductive graphene nanosheet filled the PEDOT:PSS voids on the fiber surface. The grooves and roughness of the fiber surface are consistent with those on the inner surface of the PU/graphene impregnation layer (Figures S4 and S5). Thus, we can reasonably conclude that the PEDOT:PSS fiber dented parts are just filled by the protruding parts of the PU/graphene. A similar result was also proved by Wang et al.^6^ As the temperature rises, the hydrophilic PSS will be ionized and adsorbed by the relatively hydrophilic graphene nanosheet, resulting in increased exposure of the PEDOT chain.^6^ In addition, graphene is also a temperature-sensitive material with a negative temperature coefficient;^20^ thus, the synergistic effect of the temperature-sensing property of graphene in the skin layer and more exposed PEDOT chains in the core layer improves the temperature sensitivity of the composite fibers. The sensitivity of the composite fiber can be enhanced by the increased conductivity of the PU/graphene layer. However, when the graphene content exceeds 15%, the sensitivity of the composite fiber starts decreasing. (Figure 3c). This is because the thermal conductivity of the skin layer decreases due to graphene agglomeration when the graphene content is 15% (Figure 3d). The PU/graphene layer has the highest thermal conductivity and sensitivity of the composite fiber when the graphene content is 10 wt %. Therefore, the improved sensitivity of the composite fiber compared with that of the unpackaged PEDOT:PSS fiber is the result of the synergistic effect of the electrical and thermal conductivity of the PU/graphene layer.

The temperature resolution of the composite fiber is 0.1 °C (Figure 3e), which satisfies the requirements for measuring small changes in the human body temperature. The temperature-sensing sensitivity and resolution comparison results between the composite fiber and other flexible temperature sensors used for human body temperature monitoring are shown in Figure 3f.^1,2,17,18,35−38^ More detailed data pairs are given in Table S1. The temperature-sensing sensitivity of this work reached the highest level when the resolution was 0.1 °C. In other words, the sensitivity of the composite fiber is the highest so far under the resolution (0.1 °C) that is required for the measurement of human body temperature.

Response time is another important indicator of temperature sensors. The response time of the composite fiber is 17 s when the temperature increases from 30 to 50 °C (Figure 3g), which is much faster than that of the mercurial thermometer (≥5 min) and electronic medical thermometer (≥1 min). In addition, the resistance of the composite fiber is very stable when held at a fixed temperature for 5 min (Figure 3h). The temperature-sensing sensitivity of the composite fiber is kept well after 20 cycles in 30–50 °C (Figure 3i), which indicates that the composite fiber has good cycle stability.

### Wearability of the PU/Graphene Encapsulated PEDOT:PSS Fiber and Its Fabric

**Figure 4:**
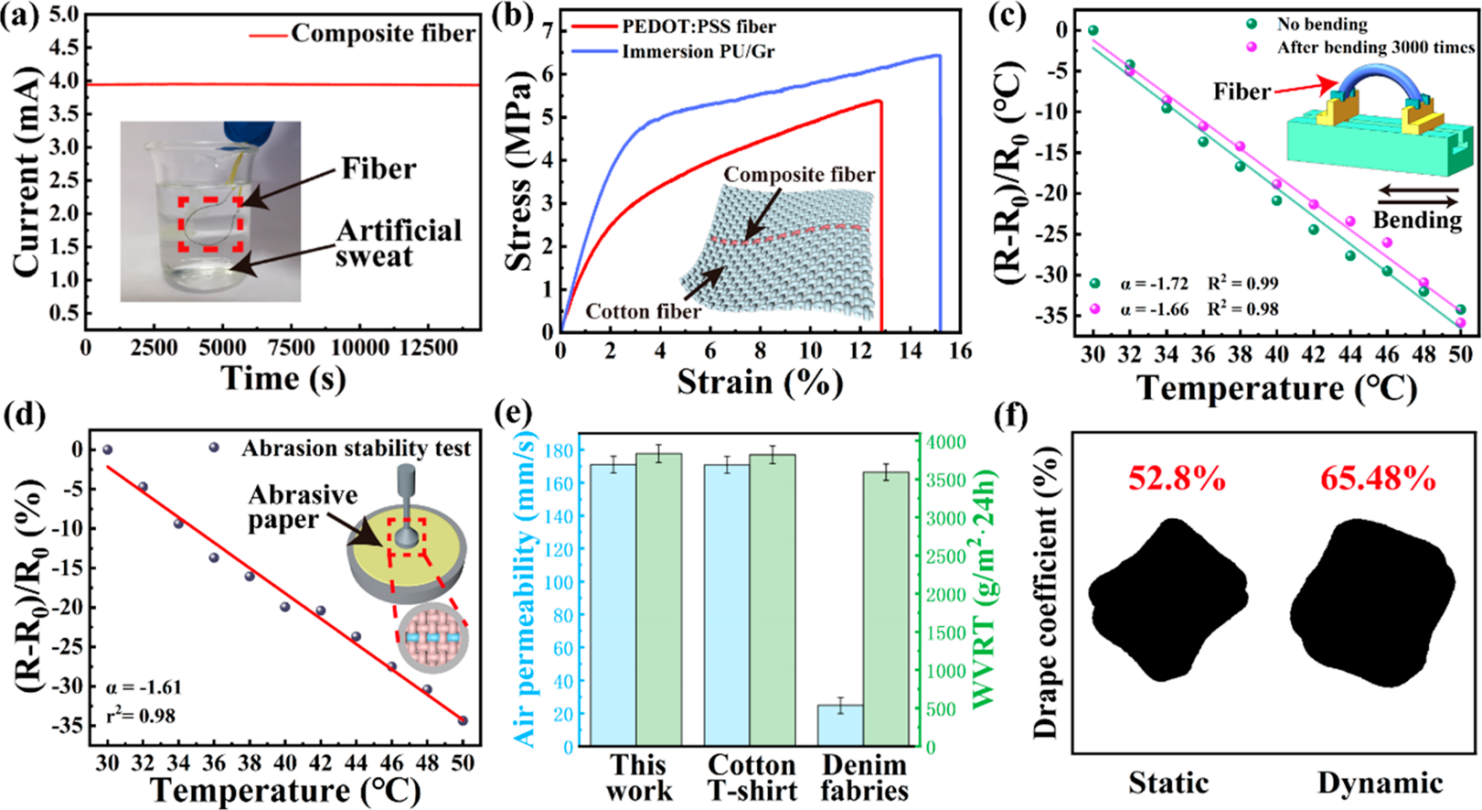
Wearability of the PU/graphene encapsulated PEDOT:PSS fiber and its fabric. (a) Current stability of the composite fiber in artificial sweat; (b) tensile properties of PEDOT:PSS fibers before and after PU/graphene encapsulation, the inset shows the schematic diagram of the fabric; (c) temperature-sensing stability of the composite fiber after bending for 3000 times; (d) temperature-sensing stability of the fabric after being treated with sandpaper for 60 times; (e) air permeability and moisture permeability of different fabrics; (f) static and dynamic drape properties of the fabric.

For practical applications in human body temperature monitoring, the wearability of the composite fiber and its fabric must be investigated. Sweating is unavoidable in daily life, especially in the underarm position. Since the composite fiber is used to measure the temperature of the human underarm, its ability to withstand sweat interference is critical. Therefore, the current changes in the composite fiber and the PEDOT:PSS fiber soaked in artificial perspiration were tested (Figure 4a). It was found that the currents of the composite fiber remained unchanged even after soaking in artificial perspiration for 14400 s (4 h), whereas the currents of the PEDOT:PSS fiber changed dramatically in response to artificial perspiration (Movie S1 and Figure S6). In addition, the sensitivity of the composite fiber shows little change when soaked in artificial perspiration (Figure S7). This indicates that the composite fiber can resist sweat interference due to the waterproof properties of the PU/graphene layer. The accuracy of temperature monitoring is not affected by sweating or exposure to environments with water, such as rain or swimming. Therefore, there is no need to worry about these factors when monitoring temperature.

In addition, if the composite fibers can be woven into clothing, they can be worn close to the body to achieve real-time monitoring of the human temperature. Figure 4b displays the stress–strain curves of the PEDOT:PSS fiber and the composite fiber. The tensile strain (15.19%) of the composite fiber is higher than the PEDOT:PSS fibers (12.84%) and is 2.17 times higher than that of cotton fibers due to the good stretchability of the PU/graphene outlayer.^39^ The resistance of the composite fiber increases slowly with the increase of tensile strain and increases by 2% when it nears failure (Figure S8). In addition, the tensile strength of the composite fiber is 6.5 MPa, which is strong enough to weave with cotton fiber to form a temperature-sensing fabric, as shown in Figure 4b; the optical picture of the fabric is shown in Figure S9. It should be noted that this composite fiber can also be woven with other commercial fibers such as polyester. Additionally, the temperature-sensing fiber must be close to the body to monitor human body temperature accurately. Cotton fiber is commonly used for intimate clothing because of its comfort and skin-friendly nature. Therefore, it is the preferred choice for weaving fabric with composite yarn in this work.

Temperature-sensing fabrics must be as durable and comfortable as clothing to ensure long-term wearing. The sensitivity (α = −1.66%/°C) of the composite fiber only decreases by 3.48% after it was bent for 3000 cycles (Figure 4c), indicating that it has good bending resistance. As shown in Figures S10 and S11, the average resistance of the composite fiber in one bending cycle is 227.00 Ω, and the standard deviation is 0.02 Ω, which indicates that bending has little effect on the resistance of the composite fiber. Figures S12 and 4d show the morphology and the temperature-sensitive performance of the fabric after different times of friction, respectively. When the fabric is rubbed with sandpaper 60 times, serious damage occurs to the other fiber, while the composite fibers remain unbroken. Upon retesting the temperature-sensitive performance of the fabrics, it was discovered that the sensitivity (α = −1.61%/°C) of the composite fiber only decreased by 6.39%. This exceptional sensitivity level still surpasses that of the most flexible temperature sensors reported today.^2,40,41^ The service life of the composite fiber is longer than that of the commercial cotton yarn, suggesting that it has the potential to detect human body temperature for a long wearing time.

Breathability, moisture permeability, and softness are very important parameters regarding fabric comfort.^14^ The breathability of the fabric compared with other common wear fabrics is displayed in Figure 4e. The air breathability of the fabric is 171.0 mm/s, which is similar to the commercial cotton T-shirt fabric (170.8 mm/s) and much higher than that of the commercial denim fabric (24.75 mm/s).^42^ The moisture permeability of the fabric is 3834 g/m^2^·24 h, which is similar to that of cotton fabrics (3819 g/m^2^·24 h) but higher than that of denim fabric (3589 g/m^2^·24 h).^42^ The drape property of a fabric is an indicator of softness or stiffness. The static drape of 52.8% and the dynamic drape of 65.48% of the fabric (Figure 4f) indicate that the fabric is soft to wear.^43^ Consequently, temperature-sensing fabrics with good durability and wear comfort can potentially monitor human body temperature in real-time while being worn for a long time.

### Applications of PU/Graphene Encapsulated PEDOT:PSS Fiber and Its Textile

To feed the data collected by the temperature-sensing fiber back to the phone in real-time, we developed a set of Bluetooth transmission systems based on Arduino Uno (Figures S13 and S14) and HC-06 Bluetooth (Figure S16; the program flow diagram and the circuit diagram of the main control chip are shown in Figure S15). The temperature-sensing element of the wearable sensor prepared in this work is the composite fiber. First, the fiber resistance at every 1 °C in the temperature range of 30–50 °C was measured. Second, the linear relationship between resistance and temperature was obtained, followed by incorporating the linear resistance–temperature function into the Arduino Uno program. Finally, Arduino Uno transmits the collected temperature data to the phone in real-time.

**Figure 5:**
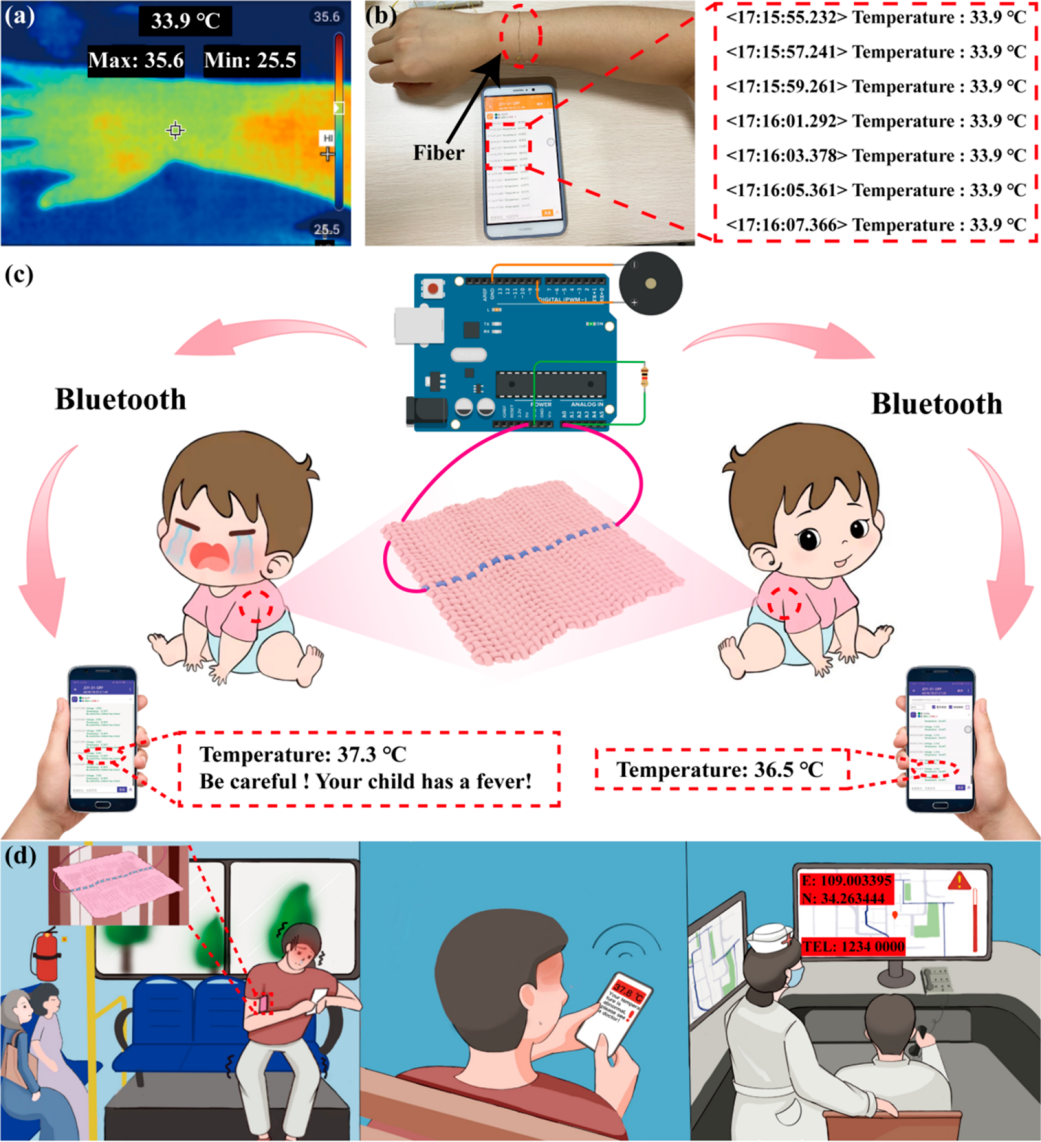
Arm temperature detected by infrared thermal imager (a) and the composite fiber (b); (c) example of the fabric with the composite fiber as a fabric-based flexible temperature sensor; (d) example of fabric-based temperature sensors for smart medical applications.

Infrared thermal imager^26,44^ was utilized simultaneously to check the sensing accuracy of the composite fiber in real applications and monitor human arm temperature. As shown in Figure 5a,b, the infrared thermal imager and message on the mobile phone display the same temperature and almost respond simultaneously, indicating that the composite fiber is accurate enough to monitor human body temperature. The recorded armpit temperature of 36.5 °C aligns with the typical temperature of an adult, which serves as proof of the accuracy of the temperature sensor created in this study (Figure S17). Figure 5c shows an example of a temperature-sensing fabric containing the composite fiber. The program was designed to notify the user when the temperature exceeds 37.3 °C. For example, when the human body temperature increases to 37.3 °C, the users will receive an alert sent to their mobile phone as “Temperature: 37.3 °C. Be careful! Your child has a fever!” (Movie S2). Since the fabric-based temperature sensor can be worn for long periods, it can monitor the human body temperature in real-time and share the temperature data with a large database by a mobile phone (Figure 5d). In case of abnormal human health, the temperature and the positioning coordinates will be uploaded to the cloud, which is conducive to disease monitoring and treatment. For example, body temperature was once an indicator for quickly recognizing COVID-19 patients. Therefore, if everyone could wear a garment containing temperature-sensing fibers, then it would help prevent and control the COVID-19 epidemic. In addition, the excellent waterproof properties of this garment allow divers and diving enthusiasts to wear it when working or playing underwater. In the event that the temperature drops because of the low water temperature underwater, an alarm will be sent in time for the guardian to respond to the potential emergency rescue.

## Conclusion

In this work, a PU/graphene-encapsulated PEDOT:PSS fiber temperature-sensor with antisweat interference, highly sensitive, and good wearability was developed in one step by combining wet spinning technology with impregnation technology. The temperature-sensing ability of the PEDOT:PSS fiber can be tuned by changing the ratio of IPA and DMSO in the coagulation bath and the washing time of deionized water. The sensitivity of the PEDOT:PSS fiber reaches an optimum value of −1.46%/°C when the ratio of IPA to DMSO is 2:1 and the washing time is 10 min. The sensitivity of the composite fiber is further improved due to the synergistic effect of the electrical and thermal conductivity of the PU/graphene layer, reaching −1.72%/°C with a linearity of 0.98. When the temperature range is 30–50 °C, the response time of the composite fiber is 17 s, which is much faster than that of the mercurial thermometer (≥5 min) and electronic medical thermometer (≥1 min). The sensing accuracy is 0.1 °C, which is consistent with the accuracy of commercial thermometers and could be measured as the minimum change in body temperature. The composite fiber is strong enough to be braided with any commercial textile yarn to form a temperature-sensitive fabric. The fabric has similar comfort and durability to that of a commercial cotton T-shirt. Besides, the sensitivity of the composite fiber is not affected by moisture such as sweat due to the excellent water-repellent property of the skin layer of the PU/graphene, so it can be arranged in the armpit position in the cloth to realize real-time monitoring of body temperature. Moreover, we developed a set of Bluetooth transmission systems based on Arduino Uno and Bluetooth HC-06 to transmit the data collected from the temperature-sensing fiber in real-time to the phone and the cloud. Therefore, the fabrics containing the temperature-sensitive fiber can be used in intelligent medical treatment and disease monitoring, which is significant for the long-term, stable, and remote monitoring of human physiological information.

## Methods

### Materials

PEDOT:PSS dispersible pellets (Zhuhai Kaiwei Optoelectronics Technology Co., Ltd.) were used as raw materials for wet spinning. The mixture of 2-propanol (IPA, Sinopharm Chemical Reagent Co., Ltd.) and dimethyl sulfoxide (DMSO, Sinopharm Chemical Reagent Co., Ltd.) was used as a coagulation bath. The mixture of PU (Huntsman, Utah, America) and graphene (Suzhou Tanfeng Technology Co., Ltd.) was used to encapsulate the PEDOT:PSS fibers. All chemicals and reagents used in this study were analytical grade and used without further purification.

### Characterization

The morphology, infrared spectra, XPS spectra, Raman spectra, tensile strength resistance, and bending fatigue resistance of the fiber was characterized by the SEM (Quanta-450-FEG, FEI, USA), FTIR spectrometer (Spotlight 400, USA), photoelectron spectrometer (ESCALAB 250Xi, USA), Raman spectrometer (InVia Qontor, UK), universal tester (Suns, China), electrochemical workstation (CHI660e, China), and the ultrasmall bending radius fixture (PR-BDM-H4E, China), respectively. The roughness of the inner surface at the junction of PEDOT:PSS fiber and PU/graphene impregnation layer was characterized by a laser confocal microscope (KC-X1000). The drying oven (DHG-9030A, China) provided different temperature environments. The fiber length for testing resistance changes during bending is 50 mm, and the fiber is bent with 180° (Figure S11) and then returned to a straight state named one bending cycle.

The woven fabric samples were prepared on a semiautomatic sample loom (SGA598, China). The drape, abrasion resistance, air permeability, water vapor permeability, and morphology of the fabric were tested by the fabric dynamic drape style instrument (YG811L, China), a martindale fabric grinder (YG401C, China), a digital air permeability meter (YG461E, China), moisture permeability tester (FX3150, Switzerland), and the three-dimensional ultradepth of field microscope (VHX-5000, Japan), respectively.

### Preparation of PEDOT:PSS and PU/Graphene Solution

First, 0.263 g of PEDOT:PSS particles was added into 5 mL of deionized (DI) water and then stirred for 30 min at 500 rpm; the resulting solution was used as the spinning solution. The PU solution was prepared at a concentration of 20 wt %. First, 80 g of PU particles was added to the solution preparation bottle. Then, 400 mL of DMF solution was poured into the bottle to uniformly dissolve the PU particles at 60 °C. After that, 5 mL of the dispersed 5, 10, and 15 wt % graphene solution were taken separately, added with 100 mL of PU solution, and evenly stirred for 60 min to produce PU/graphene solution.

## Supporting Information Available

Summary of some wearable temperature sensors and their performances; local magnification of the infrared spectrum with PEDOT:PSS particles and coagulation bath IPA/DMSO at a 2:1 ratio; conversion of PEDOT chain from benzene structure to quinone structure; Raman spectra of PEDOT:PSS fiber encapsulated with PU/graphene, PU and graphene; microscopic morphology of the exfoliated PU/graphene fiber surface and the inner surface of the PU/graphene impregnated layer; internal surface roughness of fiber surface and PU/graphene impregnation layer; stability testing of composite fibers and PEDOT: PSS fibers in artificial sweat; data graph of composite fiber test in water, sensitivity α leave unchanged; the resistance changes of the composite fiber during the stretching process; ordinary cotton fabric containing composite fibers; the resistance changes of the composite fiber during one bending cycle; fiber bending test on the FlexTest-TM-L flexible electronic test system; the optical pictures of the fabric under different friction times when the fabric is subjected to abrasion test with abrasive paper; the internal wiring structure of the Arduino board; 3D schematic of the Arduino board; circuit diagram of the main control chip of the Arduino board and program design flowchart; picture of HC-06 Bluetooth module; actual measurement at the human underarm (PDF)
Movie S1: currents of the PEDOT:PSS fiber changing dramatically in response to artificial perspiration (MP4)
Movie S2: sensor recording a temperature of 37.3 °C, triggering an alert sent to the mobile phone (MP4)
