# Supplementary material for: An Antisweat Interference and Highly Sensitive Temperature Sensor Based on Poly(3,4-ethylenedioxythiophene)–Poly(styrenesulfonate) Fiber Coated with Polyurethane/Graphene for Real-Time Monitoring of Body Temperature
Source: ACS Nano. 2023 Oct 24;17(21):21073–82. doi: 10.1021/acsnano.3c04246 (PMC10655239; doi:10.1021/acsnano.3c04246)
Supplement: Supplementary file 1 — nn3c04246_si_001.pdf [file nn3c04246_si_001.pdf]

Supporting Information

An Anti-sweat Interference and Highly Sensitive Temperature-sensor Based on Poly(3,4-ethylenedioxythiophene)-Poly(styrenesulfonate) Fiber Coated with Polyurethane/Graphene for Real-time Monitoring of Body Temperature

*Wei Fan[a, *], Tong Liu[a], Fan Wu[a], Shujuan Wang[b], Shengbo Ge[c], Yunhong Li[a], Jinlin Liu[a], Haoran Ye[c], Ruixin Lei[a], Chan Wang[a], Qiuling Che[d], Yi Li[e, *]*

[a] School of Textile Science and Engineering, Key Laboratory of Functional Textile Material and Product of Ministry of Education, Xi'an Polytechnic University, Xi'an 710048, China

[b] School of Chemistry, Xi'an Jiaotong University, Xi'an 710049, China

[c] College of Materials Science and Engineering, Nanjing Forestry University, Nanjing, Jiangsu 210037, China

[d] ANTA (China) CO., Ltd, Quanzhou 362000, China

[e] Department of Materials, University of Manchester Oxford Road, Manchester, M13 9PL, UK

*Corresponding author: fanwei@xpu.edu.cn; henry.yili@manchester.ac.uk

Table S1. Summary of some wearable temperature sensors and their performances

Materials	Sensitivity [%/℃]	Detection range	Response/ recovery time	Resolution	Method	References
Crosslinked PEDOT:PSS	-0.77	25~50 ℃	1.5 s / 6 s	——	Printing	1
PEDOT:PSS/CNT	-0.61	22~48 ℃	18 s	——	Printing	2
PEDOT:PSS/FGO-PVDF	-0.395	-10~30 ℃	40 s	1272.57 Ω/℃	Printing	3
PEDOT:PSS/cotton threads	-0.48	-50~80 ℃	——	323.4 Ω/℃	Dip dyeing	4
PEDOT:PSS/ rGO	-1.69	30~50 ℃	——	——	Aerogel	5
PEDOT: PSS/ GO	-1.09	25−100℃	18 s / 32 s	——	Drop casting	6
PEDOT:PSS/SWCNTS/PU	-0.93	20 ~ 120℃	6 s / 30 s	——	dip-coating	7
PEDOT/TPU	-0.95	20 ~ 40℃	30 s / 27 s	0.2 ℃	Situ Polymerization	8
PU/rGO	-0.8	25 ~ 49 ℃	7s / 70s	0.1 ℃	Wet-spinning	9
PAN/GO	-0.4	20 ~ 100℃	——	——	alternating coating	10
CNT/PET	-1.23	30 ~ 65℃	16.5s (30~45℃)	——	Coiling silk fiber	11
PANI NFs/PAA	-1.64	40 ~ 110℃	19.5s/20.66s	2.7℃	hydrogel	12
Pure rGO	-1.95	30 ~ 80℃	2s / 2.5s	0.4℃	Printing fiber	13
rGO	0.636	30 ~ 80℃	7s / 20s	>0.1℃	Wet-spinning	14
PVDF	0.02	35 ~ 85℃	——	2.7℃	convergence thermal drawing	15
Pt wire	0.213	20 ~ 50℃	——	0.1℃	——	16
**PEDOT:PSS/ PU/Graphene**	**-1.72**	**30 ~ 50 ℃**	**17 s / 26 s**	**0.1 ℃**	**Wet-spinning**	**This work**

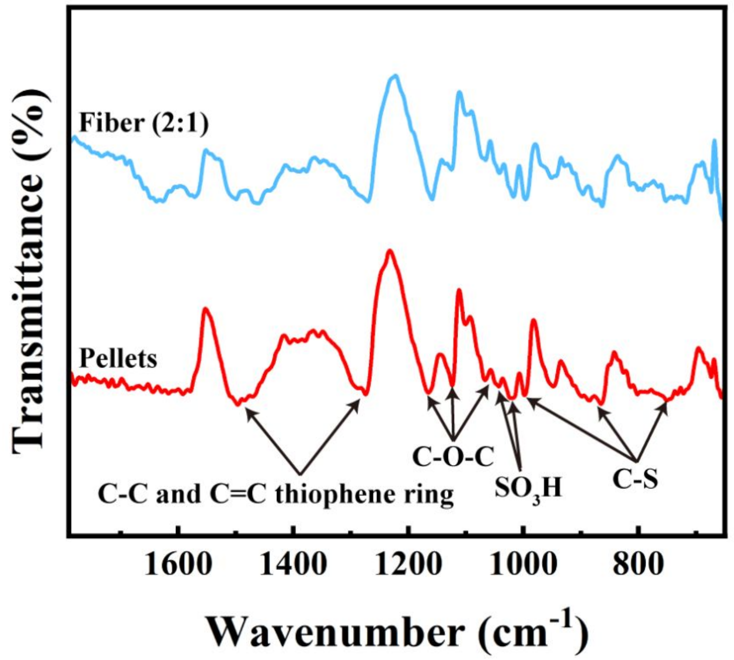

Figure S1. Local magnification of the infrared spectrum with PEDOT:PSS particles and coagulation bath IPA:DMSO at a 2:1 ratio.

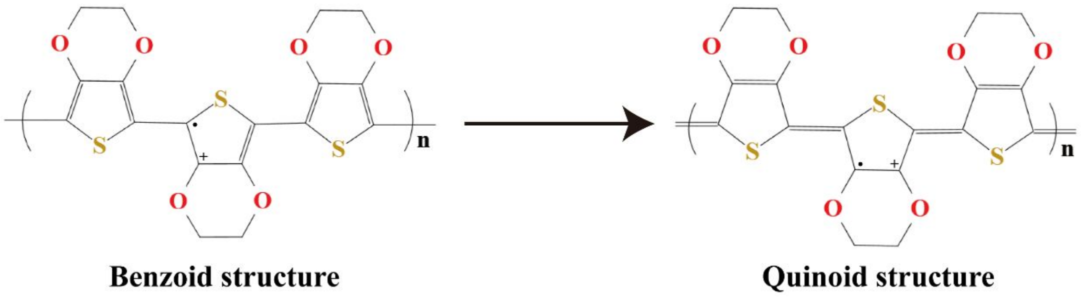

Figure S2. Conversion of PEDOT chain from benzene structure to quinone structure, "•" and "+" represent unpaired electrons and positive charges, respectively[17].

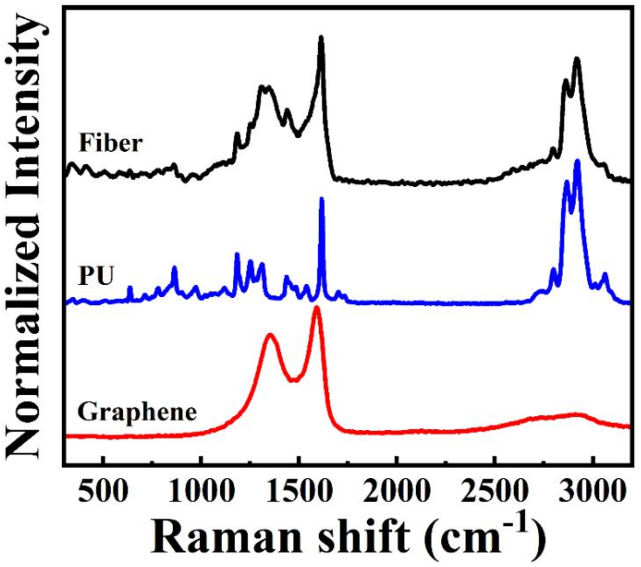

Figure S3. Raman Spectra of PEDOT:PSS fiber encapsulated with PU/Graphene, PU and Graphene.

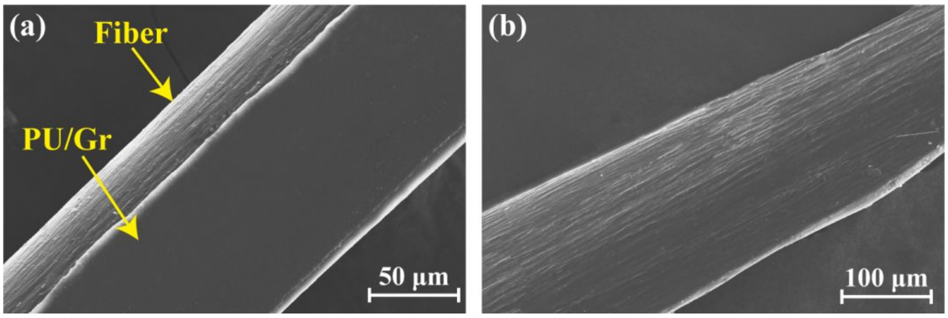

Figure S4. Microscopic morphology of the exfoliated PU/graphene fiber surface and the inner surface of the PU/graphene impregnated layer. (a) SEM image of the outer surface of the fiber and impregnation layer; (b) SEM image of the inner surface of the impregnation layer.

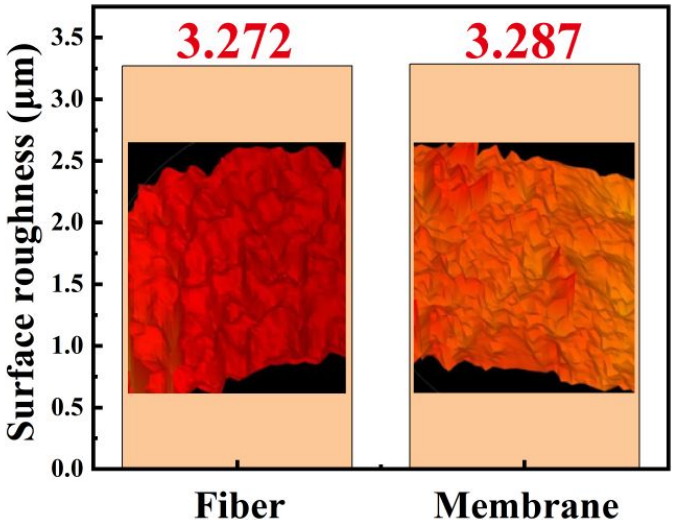

Figure S5. Internal surface roughness of fiber surface and PU/graphene impregnation layer.

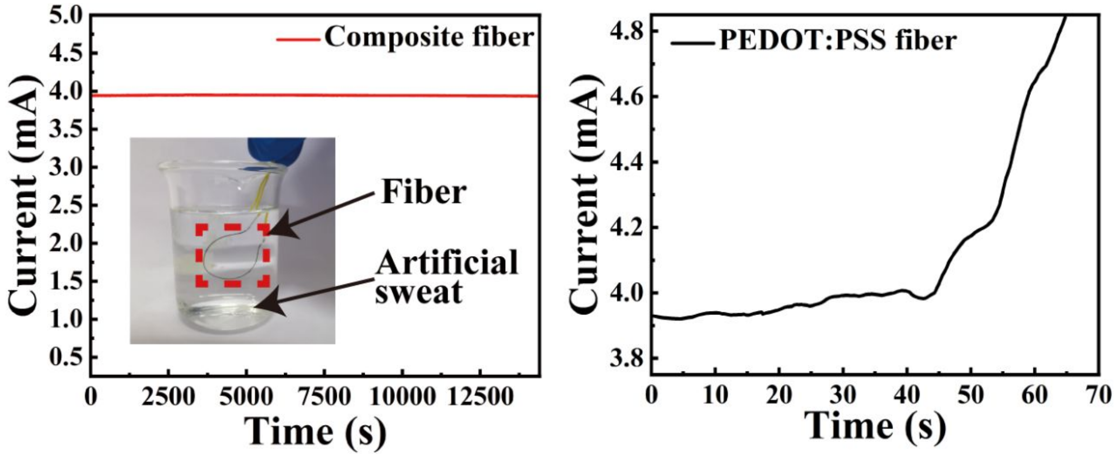

Figure S6. Stability testing of composite fibers and PEDOT: PSS fibers in artificial sweat.

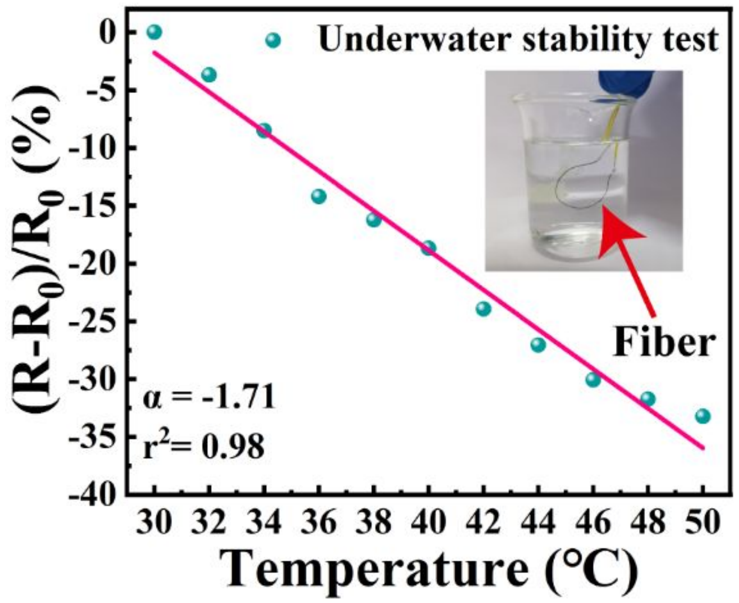

Figure S7. Data graph of composite fiber test in water, sensitivity α leave unchanged.

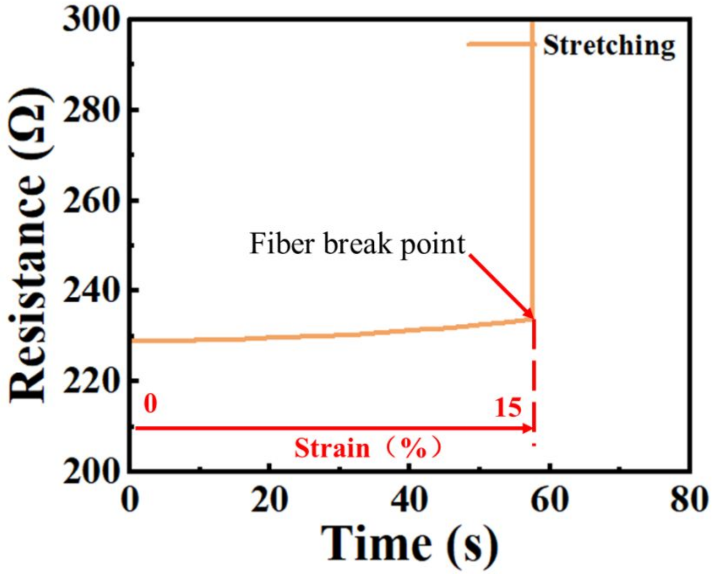

Figure S8. The resistance changes of the composite fiber during the stretching process.

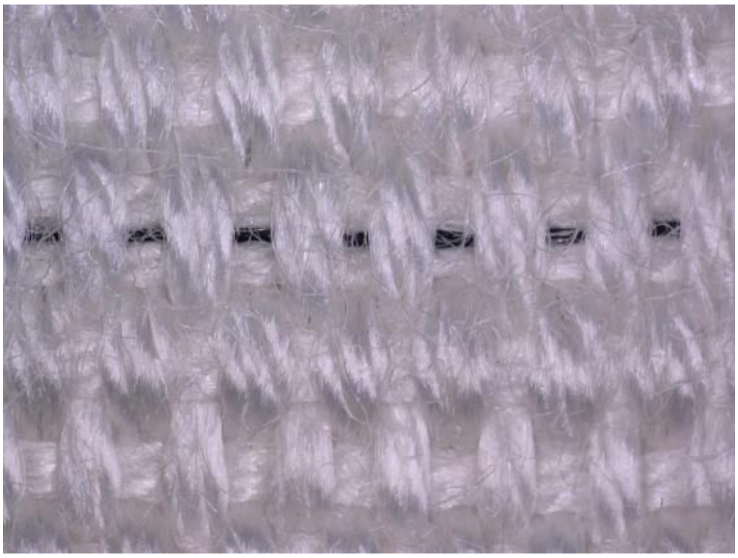

Figure S9. Ordinary cotton fabric containing composite fibers.

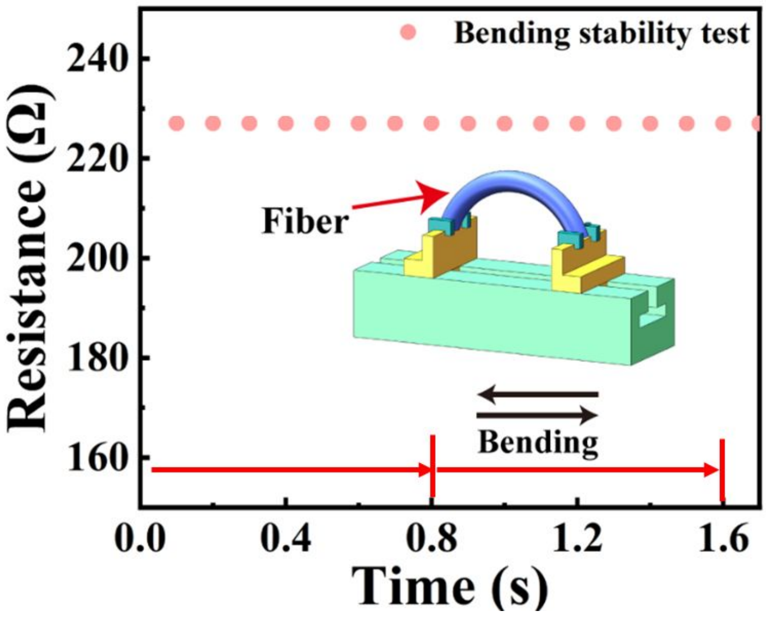

Figure S10. The resistance changes of the composite fiber during one bending cycle.

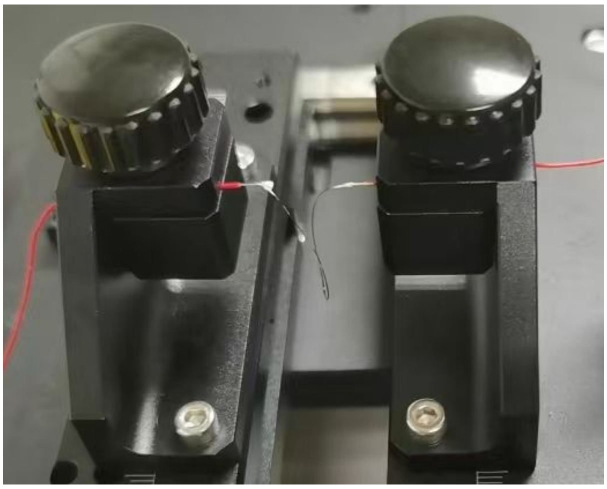

Figure S11. Fiber bending test on the FlexTest-TM-L flexible electronic test system.

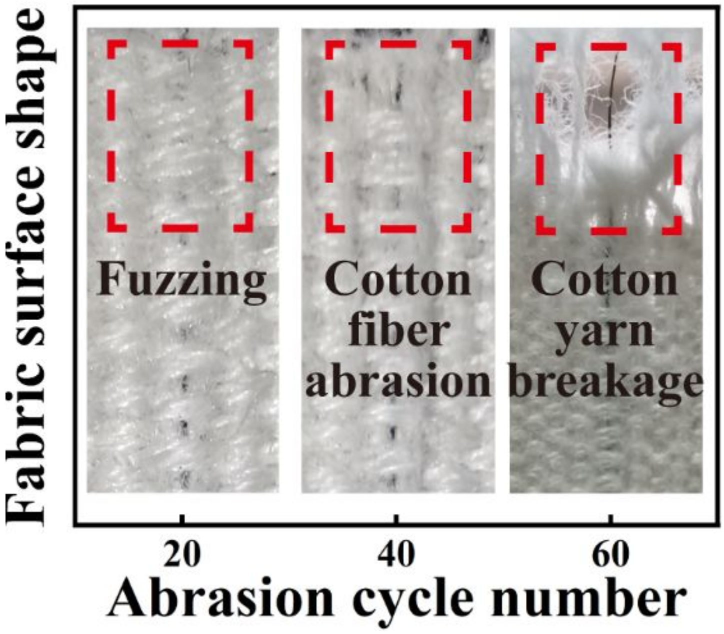

Figure S12. The optical pictures of the fabric under different friction times when the fabric is subjected to abrasion test with abrasive paper. After 60 times of friction, the cotton fiber is seriously worn, but the composite fiber in it remains intact

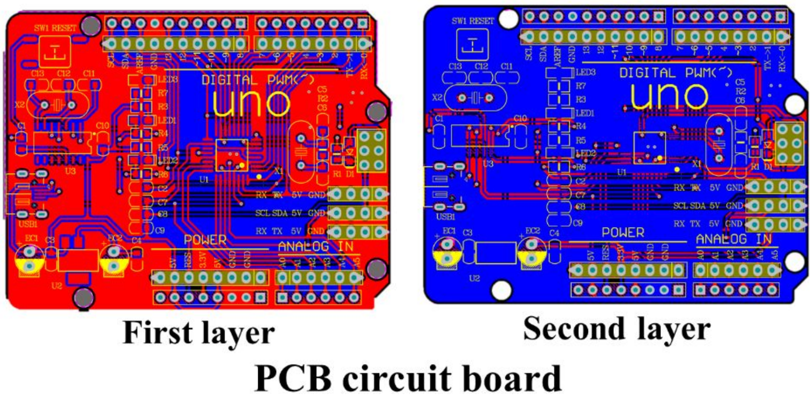

Figure S13. The internal wiring structure of the Arduino board.

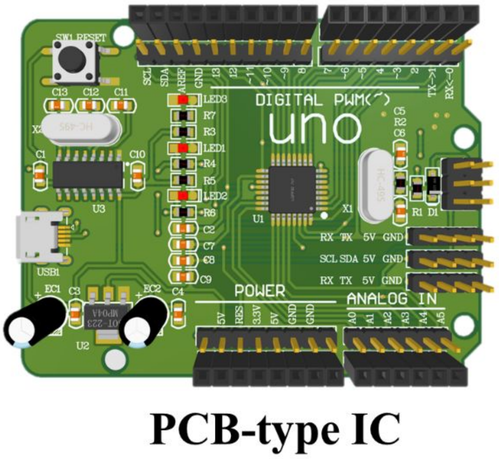

Figure S14. 3D schematic of the Arduino board.

(a)

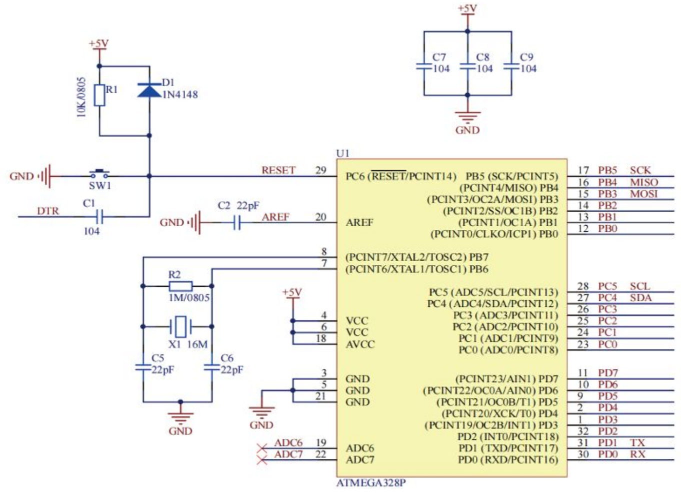

(b)

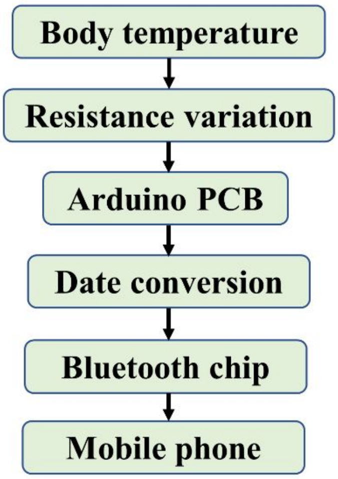

Figure S15. (a) Circuit diagram of the main control chip of the Arduino board. (b) program design flowchart.

(a)

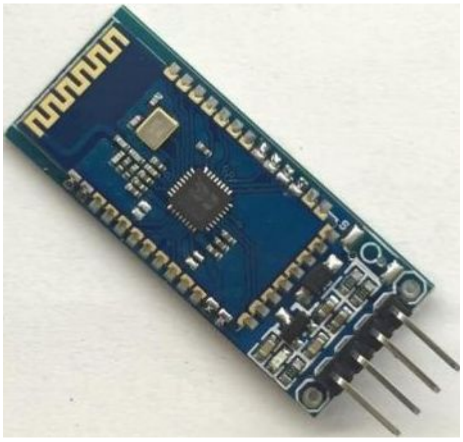

(b)

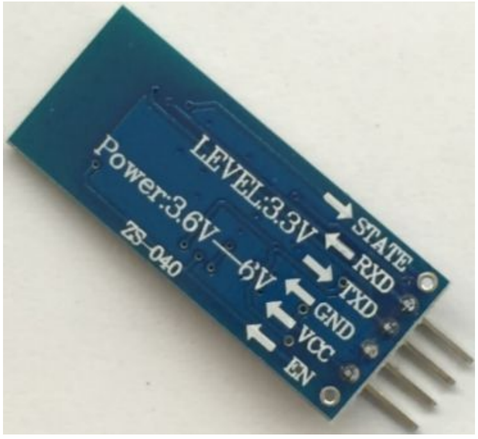

Figure S16. Picture of HC-06 Bluetooth module. (a) front (b) back. The Bluetooth module has 4 pins, which are positive VCC, negative GND, and data transmission pins TXD and RXD.

During circuit connection, both ends of the temperature sensitive fiber are connected to 5V and A0 ports of the circuit board respectively, and the resistance of 100 Ω is connected to A0 and GND terminals of the circuit board as a voltage divider to protect the circuit and more accurately measure the electrical signals at both ends of the fiber. The VCC and GND interfaces of the Bluetooth module are respectively connected to the VCC and GND of the circuit board, the RXD of the Bluetooth module is connected to the TXD of the circuit board, that is, pin 31 of the chip, and the TXD is connected to the RXD of the circuit board, that is, pin 30 of the chip. After the connection is completed, the power is turned on, and the Arduino can detect the voltage at both ends of the fiber and convert it into a temperature signal. Then the data can be transmitted to the mobile phone through the Bluetooth module.

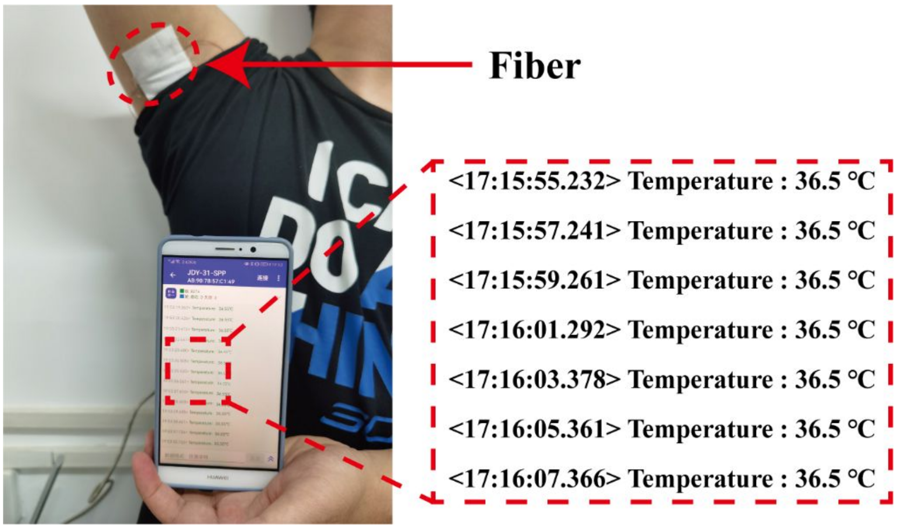

Figure S17. Actual measurement at the human underarm.

References:

(1) Wang, Y. F.; Sekine, T.; Takeda, Y.; Yokosawa, K.; Matsui, H.; Kumaki, D.; Shiba, T.; Nishikawa, T.; Tokito, S. Fully Printed PEDOT:PSS-Based Temperature Sensor with High Humidity Stability for Wireless Healthcare Monitoring. *Sci Rep*. **2020,** *10*, 2467.

(2) Honda, W.; Harada, S.; Arie, T.; Akita, S.; Takei, K. Wearable, Human-Interactive, Health-Monitoring, Wireless Devices Fabricated by Macroscale Printing Techniques. *Adv. Funct. Mater*. **2014,** *24*, 3299-3304.

(3) Maskey, B. B.; Shrestha, K.; Sun, J.; Park, H.; Park, J.; Parajuli, S.; Shrestha, S.; Jung, Y.; Ramasundaram, S.; Koirala, G. R.; Cho, G. Proving the Robustness of a PEDOT:PSS-Based Thermistor Via Functionalized Graphene Oxide-Poly(Vinylidene Fluoride) Composite Encapsulation for Food Logistics. *RSC Adv*. **2020,** *10*, 12407-12414.

(4) Lee, J. W.; Han, D. C.; Shin, H. J.; Yeom, S. H.; Ju, B. K.; Lee, W. PEDOT:PSS-Based Temperature-Detection Thread for Wearable Devices. *Sensors (Basel)*. **2018,** *18*.

(5) Zhang, F.; Hu, H.; Islam, M.; Peng, S.; Wu, S.; Lim, S.; Zhou, Y.; Wang, C.H.

Multi-Modal Strain and Temperature Sensor by Hybridizing Reduced Graphene Oxide and PEDOT:PSS. *Compos. Sci. Technol.* **2020,** *187*.

(6) Soni, M.; Bhattacharjee, M.; Ntagios, M.; Dahiya, R. Printed Temperature Sensor Based on PEDOT:PSS-Graphene Oxide Composite. *IEEE Sens. J.* **2020,** *20*, 7525-7531.

(7) Lee, J.; Kim, D. W.; Chun, S.; Song, J. H.; Yoo, E. S.; Kim, J. K.; Pang, C. Intrinsically Strain-Insensitive, Hyperelastic Temperature-Sensing Fiber with Compressed Micro-Wrinkles for Integrated Textronics. *Adv. Mater. Technol.* **2020,** *5*.

(8) Li, F.; Xue, H.; Lin, X.; Zhao, H.; Zhang, T. Wearable Temperature Sensor with High Resolution for Skin Temperature Monitoring. *ACS Appl. Mater. Interfaces*. **2022,** *14*, 43844-43852.

(9) Trung, T. Q.; Dang, T. M. L.; Ramasundaram, S.; Toi, P. T.; Park, S. Y.; Lee, N. E. A Stretchable Strain-Insensitive Temperature Sensor Based on Free-Standing Elastomeric Composite Fibers for on-Body Monitoring of Skin Temperature. *ACS Appl. Mater. Interfaces*. **2019,** *11*, 2317-2327.

(10) Ke, F.; Song, F.; Zhang, H.; Xu, J.; Wang, H.; Chen, Y. Layer-by-Layer Assembly for All-Graphene Coated Conductive Fibers toward Superior Temperature Sensitivity and Humidity Independence. *Compos. B. Eng*. **2020,** *200*.

(11) Wu, R.; Ma, L.; Hou, C.; Meng, Z.; Guo, W.; Yu, W.; Yu, R.; Hu, F.; Liu, X. Y. Silk Composite Electronic Textile Sensor for High Space Precision 2D Combo Temperature-Pressure Sensing. *Small*. **2019,** *15*, e1901558.

(12) Ge, G.; Lu, Y.; Qu, X.; Zhao, W.; Ren, Y.; Wang, W.; Wang, Q.; Huang, W.; Dong, X. Muscle-Inspired Self-Healing Hydrogels for Strain and Temperature Sensor. *ACS Nano*. **2020,** *14*, 218-228.

(13) Zhao, J.; Zhang, Y.; Huang, Y.; Xie, J.; Zhao, X.; Li, C.; Qu, J.; Zhang, Q.; Sun, J.; He, B.; Li, Q.; Lu, C.; Xu, X.; Lu, W.; Li, L.; Yao, Y. 3D Printing Fiber Electrodes for an All-Fiber Integrated Electronic Device Via Hybridization of an Asymmetric Supercapacitor and a Temperature Sensor. *Adv Sci*. **2018,** *5*, 1801114.

(14) Trung, T. Q.; Le, H. S.; Dang, T. M. L.; Ju, S.; Park, S. Y.; Lee, N. E. Freestanding, Fiber-Based, Wearable Temperature Sensor with Tunable Thermal Index for Healthcare Monitoring. *Adv. Healthcare Mater*. **2018,** *7*, e1800074.

(15) Yu, L.; Parker, S.; Xuan, H.; Zhang, Y.; Jiang, S.; Tousi, M.; Manteghi, M.; Wang, A.; Jia, X. Flexible Multi-Material Fibers for Distributed Pressure and Temperature Sensing. *Adv. Funct. Mater*. **2020,** *30*.

(16) Gu, Y.; Li, Y.; Shaw, A. Development and Performance of Flexible Temperature-Sensing Fabric. *J TEXT I*. **2021,** *113*, 2770-2777.
